# Atmospheric Pressure Plasmas in Material Science

**DOI:** 10.3390/ma14081963

**Published:** 2021-04-14

**Authors:** Sylwia Ptasińska

**Affiliations:** 1 Radiation Laboratory, University of Notre Dame, Notre Dame, IN 46556, USA; sptasins@nd.edu; 2 Department of Physics, University of Notre Dame, Notre Dame, IN 46556, USA

The long-term goal of basic material research is to develop theoretical and experimental methodologies to advance the ability to produce materials with the desired compositions and properties that can be used in various applications. There is a wide range of physical, chemical, and biological methods to synthesize and process materials, in which plasmas undoubtedly have been playing a pivotal role since Irving Langmuir first introduced its concept [1]. Moreover, nonthermal plasma’s ability to be formed under atmospheric pressure, i.e., atmospheric pressure plasma (APP), has increased scientific attention and efforts to explore it for material technologies, largely because of its low-temperature effects, high chemical reactivity, and low-cost designs. However, a better fundamental understanding of APP is still required to use it on an industrial scale. Therefore, presenting information on APP characterization and performing experimental and theoretical studies on plasmas’ effects and their interplay are indispensable for increasing our scientific understanding of complex plasma phenomena and are inseparable elements that advance our scientific achievements in the laboratory and in industrial applications. Thus, the goal of the Special Issue “Atmospheric Pressure Plasmas in Material Science” is to present recent theoretical and experimental findings on APPs that pertain to material science.

This Special Issue consists of five research papers and one review paper in which fundamental properties of APP have been explored to advance our understanding of plasma complex interactions [2,3,4] in which APP was ignited using novel plasma source designs [2,5] and has been used in a wide range of applications, particularly in nanofabrication [2,3,6], biocatalysts immobilization [7], and energy-related processes [2]. 

The review paper by Banerjee et al. provides a detailed description of APP deposition techniques for titanium dioxide films [2]. They conducted a comparison of the techniques and identified their benefits and advantages, and limitations and disadvantages. In addition, the authors specified key plasma parameters and their effects, with particular attention to structural, electronic, and optical properties of the resulting films deposited by APP. This review paper also describes the evolution of the field, the changes in the needs for specific applications—particularly for energy conversion, such as photocatalytic and photoelectrochemical processes—and the development of sophisticated APP technology. In addition to the rich literature review in this paper, it also addresses several unexplored areas and future research directions important in the plasma-based methodology for titanium dioxide deposition. 

In their paper, Tsyganov et al. [3] described their experimental investigation of nitrogen-doped graphene sheets synthesized using microwave APP with different gas precursors. They employed several techniques to characterize the material to demonstrate their method’s effectiveness in the synthesis of graphene sheets with high structural quality. Moreover, they performed an extensive optical spectroscopic analysis of APP that was beneficial in constructing and expanding their previous theoretical model. This model helped reveal the complex physical and chemical phenomena in APP. This work showed the great potential to advance this methodology for practical, industrial use of plasma-enabled production of advanced two-dimensional nanostructures.

In another study of plasma-material modification, Czylkowski et al. [5] developed and constructed a new type of microwave plasma source. This APP source’s unique feature, in comparison to others, is the shape of the plasma generated, in which it extends into the air and forms a sheet-like shape. The benefit of this shape in material processing is its larger treatment area, which allows the process to be performed in a shorter time and does not require using multiple plasma sources or performing multiple ramping of the treated area. The authors tested this source to treat a polycarbonate material. Material properties, such as wettability, surface morphology, and mechanical properties, were investigated after argon plasma treatment under several processing conditions in which the plasma power was varied. As the authors reported, their results showed promising capabilities that suggest that this novel type of APP source is an excellent contender for processing materials other than polycarbonates for large-scale industrial production.

Lapenna et al. [7] reported the effects of APP in contact with tyrosinase, one of the enzymatic catalysts, on its stability, retention, activity, and etching resistance upon plasma exposure under several chemical environments and processing conditions. In addition, they explored the possibility of enzyme overcoating by a plasma-deposited hydrocarbon polymer film to improve tyrosine immobilization. A comparison between the results obtained for tyrosinase and glucose oxidase, an enzyme studied previously under identical plasma processing conditions, was also provided and showed that the two-step immobilization procedure proposed, in which the enzyme is deposited on a support first and coated subsequently with a polymer layer using APP, can be exploited universally. Therefore, as an alternative immobilization strategy, this methodology contributes to broadening APP functionalities in materials processing.

The work of Cejas et al. presents a numerical model that they developed to describe and simulate discharges in APPs [4]. A peculiar feature of this model is that it takes into account associative ionization with the participation of excited atoms, which other air discharge models do not typically consider. They tested the model under several experimental conditions and found that its predicted outcomes agreed well with the data observed. This work demonstrates that including more relevant reactions in theoretical and computational models is a definitive step that improves our understanding of APP complex interactions and is relevant to many practical applications, including plasma material processing.

Another study also developed numerical methodologies that were used in three-dimensional models of fluid fields and their effects on plasma generation and fouling elimination [6]. Bai et al. [6] performed their simulations with APPs that were launched into the open air through different inlet styles of central and sheath gases. The authors also synthesized nanopowders using the same design and conditions as the simulations, by which they verified the results obtained from their modeling experimentally. Based upon their findings, they proposed a strategy that can be adopted to construct a new APP source to avoid fouling, which is one of the main problems encountered during material synthesis.

This Special Issue is a collection of papers that demonstrates the importance and necessity of addressing the challenge of understanding APP processes with materials better through different theoretical and experimental methodologies. These works presented original and novel solutions with which studies performed in lab-based settings can contribute greatly to industrial technologies to synthesize and manufacture important materials.
